# Reconstructing the complex architecture of the genome with molecular scissors: applying genome editing technology in precision medicine

**DOI:** 10.3389/fgeed.2026.1762449

**Published:** 2026-04-30

**Authors:** Md Nur Amin Khan, Rohit Das, Pooja Barik, Somasundaram Arumugam, Shiladitya Chattopadhyay

**Affiliations:** 1 Department of Biopharmaceuticals, National Institute of Pharmaceutical Education and Research Kolkata (NIPER Kolkata), Kolkata, India; 2 Department of Pharmacology and Toxicology, National Institute of Pharmaceutical Education and Research Kolkata (NIPER Kolkata), Kolkata, India

**Keywords:** CRISPR, genome editing, precision medicine, TALEN, ZFN

## Abstract

Our past, present, and future are governed by the grand design of the genetic blueprint, which was mapped more than two decades back. While the eloquent design of our genome results from millions of years of evolution and has reached a near-perfect stage, unwanted flaws and mistakes in individual genomes can make their life miserable and worth intervention. The advent of technologies to manipulate the grand design and make it congenial for the individual has given new hope. Here, we discuss how gene editing technologies have progressed and how some of the technologies have become indispensable gears in precision medicine.

## 1 Introduction

Gene editing technology, or genome editing, creates specific modifications to target genes. Gene therapy is a treatment approach that uses gene editing techniques to find a cure for many diseases. High-throughput DNA sequencing and improved computational methods have made it possible to decode genome sequences from various organisms, including humans. Sequential editing, such as deletion or individual alteration, is required to understand gene functions and examine the resulting mutant phenotypes. Programmable nucleases exploit natural DNA repair processes to create specific sequence changes. These approaches produce double-strand breaks in the genome at predetermined locations using specifically manipulated nucleases (Zheng et al., 2024). Cells can spontaneously repair DSBs introduced into the genome, primarily by non-homologous end-joining or homologous recombination processes. Innovations in high-throughput DNA sequencing and genome editing have greatly influenced work on model organisms, evolutionary studies, food organism improvement, and clinical research and intervention. CRISPR-Cas genome editing methods have gained extensive acceptance in the scientific community and are increasingly finding use in the commercial sector (Huang and Zhou, 2021). To keep us focused on the big picture, this perspective goes over the history of genome editing platforms and addresses some contemporary technical difficulties. Restriction endonucleases, the molecular scissors that revolutionised molecular biology and biotechnology in the 1970s by enabling cloning, are ineffective for delivering a specific chromosomal DSB because they recognise minimal DNA sites (typically 4-8 bp), which are overrepresented in most human genomes (Khalil, 2020). Due to their unique identity, meganucleases could not adapt to the desired target areas. The necessity to induce precise DSBs has hindered the development of HDR technology for gene modification in plant and mammalian cells, including human cells. The success of three novel gene editing technologies (Table 1)—zinc finger nucleases (ZFNs), transcription activator-like effector nucleases (TALENs), and the clustered regularly interspaced short palindromic repeats (CRISPR)—CRISPR-associated (Cas9) system—has recently regenerated interest in gene therapy (Kim and Kim, 2014). These technologies possess significant healing properties because they significantly reduce the possibility of off-target mutagenesis (LaFountaine et al., 2015). The capacity of these enzymes to cause genome-specific DNA cleavage, which may be repaired (by endogenous mechanisms), makes them useful in research, medicine, and biotechnology for high-precision genome editing (Kim and Kim, 2014). This review provides insights into the origins of programmable nucleases (ZFNs, TALENs, and RNA-guided CRISPR-Cas9) for genetic alteration, covers key points, and investigates their biological and therapeutic applications, specifically in precision medicine (Metje-Sprink et al., 2019). Although these three genome editing technologies have been the focus of numerous recent primary research papers and reviews, we will give a thorough overview of their mechanisms, diverse applications, therapeutic delivery strategies, present drawbacks, and future directions in this manuscript.

**TABLE 1:** Difference between ZFN, TALEN, and CRISPR.

Attributes	ZFN	CRISPR/Cas9	TALEN
Site of recognition	Zinc finger protein	Single-stranded guide RNA	Region of the TALE protein
Enzyme required	Fok1 nuclease	Cas9 nuclease	Fok1 nuclease
Target length	Typically, 9–18 bp per ZFN monomer, 18–36 bp per ZFN pair	Typically, 20 bp guide sequence + PAM sequence	Typically, 14–20 bp per TALEN monomer, 28–40 bp per TALEN pair
Limitation	Difficult to target non-G-rich sites	The targeted site must precede a PAM sequence	5ʹ targeted base must be a T for each TALEN monomer

## 2 Historical perspective

The beginning of gene therapy can be marked in the 1960s with the advent of recombinant DNA (rDNA) technology, which later expanded to incorporate comprehensive genetic engineering techniques, including the use of viral vectors (Tamura and Toda, 2020). The concept of gene augmentation or gene replacement therapy emerged after DNA was recognised as a transformative substance capable of altering phenotypes and carrying genetic information. In 1968, Rogers et al. demonstrated that viruses could introduce exogenous DNA or transgenic DNA into cells, providing the first proof of virus-mediated gene transfer (Malech et al., 2022).

**FIGURE 1:**
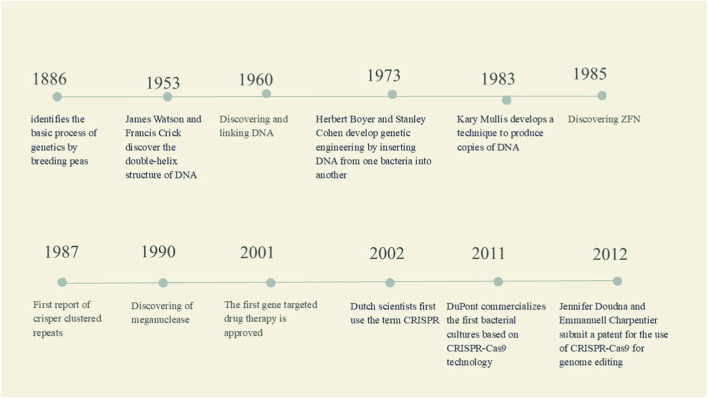
History Timeline for the major milestones in the genetic engineering and gene editing technologies.

By 1980, gene delivery systems such as needle injection, micro-injection, ballistic DNA, electroporation, sonoporation, and magnetoporation had been developed, alongside materials like calcium phosphate, silica, and gold. Non-viral vectors, which use physical or chemical methods for delivery, are still being explored for large-scale production but often result in lower transfection rates, limiting their therapeutic efficacy (Mellott et al., 2012). In 1989, the NIH’s Recombinant DNA Advisory Committee introduced the first guidelines for gene therapy clinical trials. Since then, over 1900 clinical trials have been conducted, utilising various gene-editing techniques. The FDA approved retroviral vectors as viral gene delivery systems, and key gene-editing technologies emerged, including zinc-finger nucleases (ZFNs) in 1985, transcription activator-like effector nucleases (TALENs) in 1987, and CRISPR/Cas9 in 2011(Figure 1) (Wivel, 2014).

Initial studies focused on oncogenic viruses and their ability to integrate into mammalian genomes, which led to engineering viruses to deliver genetic material, especially to human hematopoietic stem cells (HSCs). Successful gene therapy needs efficient tools for procuring, purifying, and culturing HSCs, as well as safe vectors for transduction (Bin Umair et al., 2022). The field has evolved through key advances, including the understanding of the human genome, the development of critical materials and vectors, and improvements in stem cell collection, all contributing to both successes and challenges in gene therapy.

## 3 Methods used for gene editing

**FIGURE 2:**
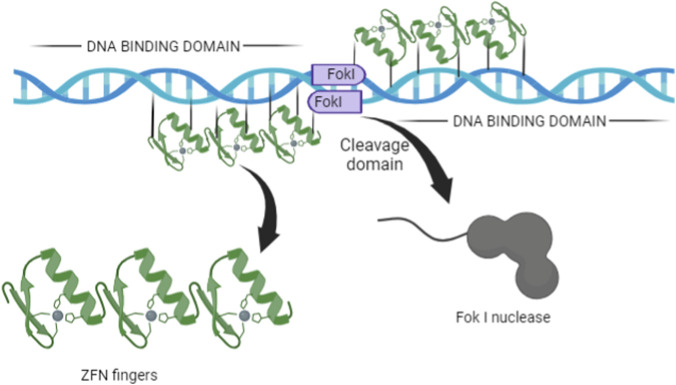
This schematic diagram illustrates Zinc-Finger Nucleases (ZFNs). Each ZFN is a chimeric protein composed of a sequence-specific DNA-binding domain, comprising multiple zinc finger repeats (green structures with grey spheres representing zinc ions), fused to a non-specific DNA-cleavage domain derived from the FokI restriction endonuclease (purple rectangles).

**FIGURE 3:**
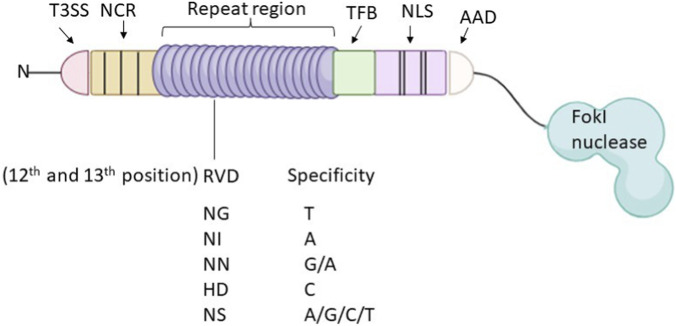
TALE contains central repeat regions of highly constant variables of 33–35 amino acids, and repeat-variable di-residues determine the specificity at positions 12 and 13. The N-terminal of TALE contains a type III secretion signal (T3SS) and four non-canonical repeats (NCR); the C-terminal contains a transcription factor binding site (TFB), two nuclear localization signals (NLS), and an acidic activation domain (AAD).

**FIGURE 4:**
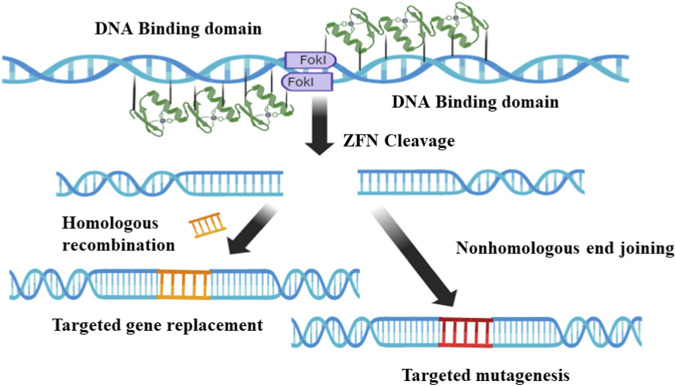
This schematic illustrates the targeted DNA cleavage mechanism by Zinc-Finger Nucleases (ZFNs). Each ZFN recognizes and binds to a specific target half-site on opposite strands of the DNA double helix. This precise binding brings the C-terminal FokI cleavage domains of the two ZFNs into close proximity. Proper spatial arrangement, the two FokI monomers dimerize, forming an enzymatically active complex (indicated by the Cleavage domain). This dimerization is crucial for the catalytic activity of FokI, as each monomer possesses only a single catalytic site. The activated FokI dimer then introduces a double-strand break (DSB) at a defined position outside the ZFN binding sites, leading to the targeted cleavage of the DNA molecule. This targeted DSB is the initiating event for subsequent cellular DNA repair pathways, enabling precise genome editing.

**FIGURE 5:**
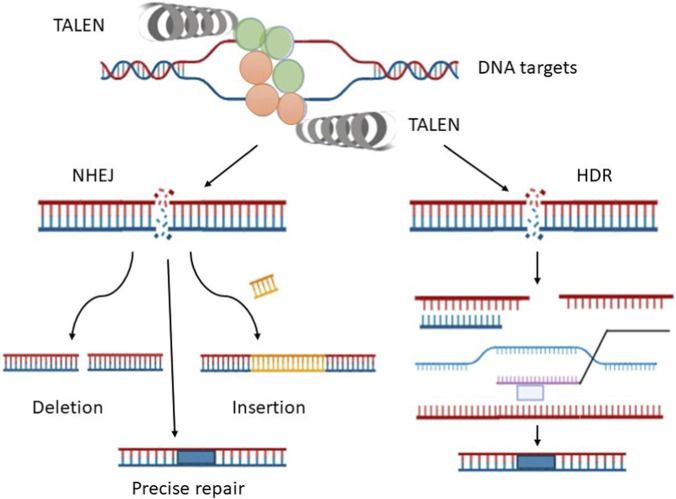
This schematic illustrates the targeted DNA cleavage mechanism of TALEN. The endonuclease activity of FokI cleaves the targeted DNA into double-stranded breaks (DSB). The DSB is repaired by two mechanisms: non-homologous end joining (NHEJ) and homology-directed repair (HDR). In NHEJ, the double-strand break is either precisely repaired or repaired by the deletion of enzymes or by the insertion of additional DNA. In HDR, the DNA is removed by repair enzymes; the 3′ end of the damaged DNA contains donor DNA and acts as a primer, enabling the repair enzyme to easily copy the information from the donor DNA. Additionally, the break is repaired, polymerized, and ligated.

**FIGURE 6:**
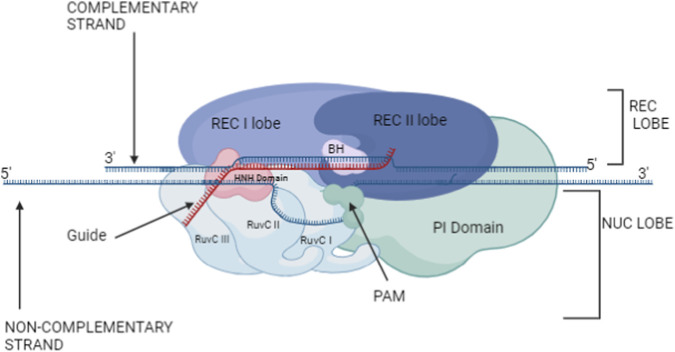
Structural organization of the CRISPR-Cas9 complex, highlighting the molecular mechanism by Cas9 introduces site- specific double -strand breaks, directed by the gRNA and PAM interaction. The Cas9 protein comprises of two main lobs: the Recognition lobe (REC) which includes the REC I and REC II domains which is accountable for binding to the guide RNA (gRNA) and sensing the target DNA and the Nuclease lobe (NUC) comprises the RuvC (I, II and III) domains, the HNH domain and the PI domain, Which are accountable and required for cleavage of the DNA strands such as the HNH domain cleaves the complementary strand while the RuvC domains cleave the non-complementary strand. Here, the guide RNA in Red colour is shown hybridizing to the Complementary strand, which is in blue colour; simultaneously, the non-complementary strand is getting displaced. The PI domain is compulsory for the recognition of the target DNA utilizing the protospacer Adjacent Motif (PAM) sequence, while the Bridge Helix (BH) is necessary for coordinating the DNA binding and cleavage.

Zinc Finger Nucleases (ZFNs), Transcription Activator-Like Effector Nucleases (TALENs), and Clustered Regularly Interspaced Short Palindromic Repeats (CRISPR) are the most widely used gene-editing tools (Gaj et al., 2013). These technologies employ restriction enzymes to create double-stranded breaks in DNA at specific sites (Figures 4, 5), guided by homologous binding proteins or RNA (LaFountaine et al., 2015). Various Target-Specific nucleases have been technologically advanced from the CRISPR-associated system found in bacteria. These include ZFNs, TALENs, and RNA-guided engineered nucleases (RGENs), which enable precise gene modifications in both cultured cells and whole animals and plants (Kim and Kim, 2014). ZFNs and TALENs are first- and second-generation technologies that utilise nucleases with both DNA-binding and cleavage domains (Figures 2, 3). These methods modify the DNA-binding domain to target sequences or motifs, which are then cleaved by the nuclease domain. However, the broader application of these first- and second-generation technologies is limited by several factors (Gaj et al., 2013). The CRISPR-Cas system, modelled after the adaptive immune system of bacteria, was developed to address these limitations. CRISPR gene editing requires two main components: a Cas protein and a transactivating single-guide RNA (sgRNA). Cas9, a nuclease, binds to the sgRNA and is directed to a specific genomic locus, called the Protospacer Adjacent Motif (PAM) site, via a 20-base pair nucleotide sequence within the sgRNA (Figure 6) (Xu and Li, 2020). Comparing the three major gene editing tools—mainly ZFNs, TALENs, and CRISPR—they are widely used in genetic engineering. Table 1 highlights the major differences between the technologies.

### 3.1 ZFN (zinc finger nuclease)

Zinc-finger nucleases (ZFNs) are engineered enzymes that target specific genes by combining a zinc-finger DNA-binding domain with a DNA-cleavage domain. ZFN-induced double-strand breaks typically activate the cell’s DNA repair mechanisms, resulting in targeted mutagenesis and gene replacement (Figure 4) at high frequencies (Carroll, 2011). In recent years, (Figure 1) ZFNs have become one of the most flexible and impactful strategies for homologous recombination due to their distinct DNA-binding and cleavage abilities.

ZFNs are created by fusing a zinc finger protein (ZFP) to the cleavage domain of the FokI restriction enzyme. The FokI enzyme, a natural type IIS restriction enzyme, has unique binding and cleavage activities, which allow for targeted gene replacement or mutation in double-stranded DNA. Each zinc finger in a ZFN interacts with three base pairs of DNA. A typical ZFN consists of multiple fingers, enabling it to recognise longer DNA sequences (Urnov et al., 2010). For instance, a three-finger ZFN can bind to a total of 9 base pairs, while arrays of fingers can target sequences ranging from 18 to 36 base pairs, depending on the number of fingers used.

The FokI cleavage domain, fused to the zinc finger domains, requires dimerization to cleave DNA (Urnov et al., 2010; Paschon et al., 2019). This means that two ZFN molecules must bind to their respective target sites on opposite DNA strands (Figure 2), forming an active dimer that induces a double-strand break (DSB) (Carroll, 2011). For effective cleavage, the two ZFNs must recognise sequences that are adjacent or closely located on the target DNA. The typical design requires that the 5′ edges of the binding sites be separated by five to seven base pairs, optimizing dimerization and cleavage by the FokI domain. When both ZFNs attach their respective sequences, the resulting high local concentration enhances the likelihood of FokI domain dimerization, thereby promoting DNA cleavage. ZFNs have therapeutic potential, such as disrupting the expression of the HIV host co-receptor CCR5 gene (Cradick et al., 2010; Liu et al., 2017).

### 3.2 TALEN (transcription activator-like effector nuclease)

TALEN is a versatile genome-editing technology that enables precise modifications to the genome by inducing double-strand breaks (DSBs) at specific sites. This approach can either directly modify the DNA or leverage the cell’s repair mechanisms to achieve the desired genetic changes. TALEN utilises engineered nucleases consisting of a sequence-specific DNA-binding domain derived from transcription activator-like effectors (TALEs), which are linked to a FokI nuclease domain (Wright et al., 2014). To cleave DNA, the FokI domain must dimerise, which requires two TALENs to bind to adjacent target sites on opposite DNA strands, typically 12 to 25 base pairs apart. The binding of these TALEN pairs facilitates dimerization, leading to efficient cleavage. TALENs share structural similarities with ZFNs, including the nonspecific FokI nuclease and DNA-binding domain, both of which also induce targeted double-strand breaks (DSBs) (Figure 3). However, genome editing using nuclease-mediated methods was historically difficult to accomplish (Becker and Boch, 2021).

### 3.3 CRISPR (clustered regularly interspaced short palindromic repeats)

In response to the threat of bacteriophages, prokaryotic organisms have evolved several defence mechanisms. Among these, the CRISPR-Cas system has emerged as remarkably significant (Bhatia and Pooja, 2023). This defence system is encoded by operons and exhibits a remarkably diverse architecture (Figure 6) (Makarova et al., 2011). The RNA transcribed from CRISPR protects prokaryotes against foreign DNA, such as viruses, conjugative plasmids, and bacteriophages. Cas (CRISPR-associated protein) is crucial for this protection and is believed to mediate the cleavage of foreign DNA through its N-terminal histidine-aspartate (HD) domain (Mulepati and Bailey, 2011).

The CRISPR/Cas system operates in three primary stages: adaptation, expression, and interference. During adaptation, foreign DNA fragments are inserted into the CRISPR locus of the host chromosome with the assistance of Cas1 and Cas2 proteins. In the expression stage, the guide RNA (gRNA) is produced and undergoes maturation from the transcribed pre-gRNA. Finally, in the interference stage, the foreign DNA is cleaved by the Cas protein bound to the mature gRNA (Moon et al., 2019).

Thus, CRISPR, along with its associated Cas protein, not only confers adaptive immunity to prokaryotic organisms but has also gained historic popularity among researchers and in therapeutic applications as a powerful gene-editing tool. The CRISPR/Cas9 system, in particular, has become a versatile and user-friendly editing tool, offering an advantage over TALEN.

## 4 Mechanism of action of the genome editing tools

### 4.1 ZFN

ZFNs are constructed by combining a site-specific DNA-binding domain, derived from the zinc finger, with a non-sequence-specific cleavage domain. The DNA recognition domains, Cys2 and His2, are the most common types of DNA-binding domains, with each ZF unit recognising 3 base pairs (bps) of DNA. The target sequence recognition of ZFNs is determined by three main factors. Those are (i) the amino acid motif, (ii) the number of fingers, and (iii) the interaction with the nuclease domain (Figure 2). The modular structure of ZFNs allows for the individual optimisation of the DNA-binding and enzymatic domains, enabling researchers to create new configurations that are both specific and have affinities suitable for genomic modification.

ZFNs, which combine the specially developed Cys2-His2 zinc-finger molecule with the FokI cleavage domain, were the first targeting nucleases to gain widespread application. These dimers recognise a specific “half-site” sequence of 9–18 DNA base pairs (bps) through their zinc-finger DNA-binding domain. The FokI cleavage region mediates ZFN protein dimer formation by cutting DNA at a 5 to 7-bp spacer nucleotide separating two adjacent zinc-finger binding sites. ZFNs typically consist of three or four zinc-finger domains, each comprising around 30 amino acid residues assembled into two anti-parallel beta sheets opposing an alpha helix. Each ZF unit recognises 3 bps of DNA and generates base-specific interactions through its α-helix residues that interrelate with the DNA’s major groove. The detection molecules within the α-helical domain frequently deal with the DNA. The FokI restriction endonuclease enzyme then forms the cleavage domain, which cuts the DNA (Figure 4). When eukaryotic cells are introduced to DNA cleavage by ZFNs, double-strand breaks (DSBs) are induced at specific genomic loci, leading to the desired changes through the subsequent endogenous repair pathways, either non-homologous end joining (NHEJ) or homology-directed repair (HDR).

### 4.2 TALEN

TALEs (Transcription Activator-Like Effectors) are type III molecular effector proteins derived from the Gram-negative plant pathogen *Xanthomonas* species. These proteins induce gene expression by binding to target promoters in the nucleus. TALE proteins have three functional domains (Figure 3).

N-terminal domain: This domain is responsible for the bacterial secretion signal and non-specific DNA-binding activity, which is crucial for protein-DNA affinity. It contains a type III secretion signal (T3SS) and four non-canonical repeats (NCR).

C-terminal domain: This domain includes a transcription factor binding site (TFB), which facilitates interaction with the plant transcription factor IIA. It also contains two functional nuclear localisation signals (NLS) and an acidic activation domain (AAD).

Central repeats: These consist of highly conserved 33–35 amino acid-long tandem repeats.

The DNA specificity of a TALE or TALEN is determined by two amino acids at positions 12 and 13 of each repeat, known as repeat-variable di-residues (RVDs). While the 13th amino acid establishes nucleotide selectivity by interfacing with the DNA’s main groove, the 12th amino acid strengthens the loop by creating hydrogen bonds with its protein backbone. Specific combinations of RVDs have been shown to recognise particular nucleotides, allowing TALEs and TALENs to target single nucleotides, pairs, triplets, or even all four nucleotides. The DNA-binding specificity of TALEs and TALENs can be modified by rearranging the repeats (Figure 3). The way the repetitions interact with the DNA bases to form a naturally right-handed helical around the DNA is revealed by the crystalline configuration of TALEs. Two alpha helices are contributed by each repeat, and they are joined by RVDs found in the loop regions. Four non-canonical repeats, designated −3, −2, −1, and 0, are found in TALEs; repeat −1 recognises an extra thymine base. One of the unique features of TALEs is their ability to use naturally occurring aberrant repeat lengths. These proteins can recognise target sequences as well as 1-bp deletions when aberrant repeats are included in the repeat array.

Another distinctive feature of TALEs is their ability to discriminate between different methylation states of nucleotides. The normal RVD, HD, recognises cytosine but does not recognise 5-methylcytosine. However, RVD N* can recognise both methylated and non-methylated cytosines. This property makes TALEs valuable for methylation-dependent gene manipulation, allowing them to differentiate between two target sequences with a single methylated nucleotide. TALEs also utilise a “hopping and sliding” method to detect DNA target sites. Sliding is the process by which the cellular protein revolves around the DNA to promote binding, whereas hopping is the process by which the protein de- and re-associates with DNA.

#### 4.2.1 Mechanism

Compared to earlier nuclease types, TALENs represent a groundbreaking class of engineered cleavage enzymes that are easier to construct and more precise in cleaving specific locations within a DNA sequence (Ding et al., 2013). TALENs combine the restriction endonuclease’s catalytic domain and the TALE DNA-binding domain FokI, binding to either the N-terminal or C-terminal end of the TALE. TALENs are arranged in pairs, oriented oppositely on either side of double-stranded DNA, with an optimal spacer sequence between them. The length of the C-terminal domain depends on the optimal spacer length. The longer DNA target site and the requirement for thymine at the 5′ end of the target result in increased specificity and reduced exonuclease activity.

The FokI nuclease cleaves the target DNA site, acting as a DNA-damaging agent and creating a double-strand break (DSB). DNA repair can then occur through either non-homologous end joining (NHEJ) or homology-directed repair (HDR). In NHEJ, the ends of the DSB are rejoined without the need for a DNA template, often leading to small insertions or deletions, single-strand annealing (SSA), microhomology-mediated end joining (MMEJ), or restoration of the original sequence (Figure 5). In contrast, HDR allows for precise repair by using a donor DNA molecule as a template, guided by extended regions of homology between the ends of the DSB. HDR requires a free 3′ end, which serves as a primer for copying information from the donor DNA until polymerisation is complete and the broken ends are filled.

#### 4.2.2 Delivery

The transfection efficiency for delivery methods in animals is generally higher than in plants. Various delivery methods are employed, including: 1. Physical methods: (i) Biolistic transformation (also known as the gene gun): This method involves coating TALEN DNA with gold or tungsten particles, which are then shot into plant cells. (ii) Microinjection: TALENs are injected directly into fertilized eggs or embryos of zebrafish, frogs, and mice. (iii) Electroporation: An electrical field is applied to increase the permeability of the cell membrane, allowing TALENs to enter the cells. 2. Bacterial-based delivery: TALEN constructs are incorporated into *Agrobacterium tumefaciens*, which then transfers the constructs into plant cells during co-cultivation. 3. Viral-based delivery: Modified viruses, such as adenoviruses, lentiviruses, and baculoviruses, are used to deliver TALENs into dividing cells for *in vivo* applications. 4. Chemical methods: Protoplasts from plant cells are transformed with TALEN constructs through a chemical treatment using polyethylene glycol (PEG).

### 4.3 CRISPR

There are two main categories of the CRISPR-Cas system, which are distinguished by the organisation of their effector modules (Table 2) (Mohanraju et al., 1979). The classification is based on a two-step approach: 1) identifying all the Cas genes in each CRISPR-Cas locus, and 2) determining the signature genes and distinctive gene structures that enable the assignment of these loci to specific types and subtypes (Ma et al., 2015). The primary difference between the Class 1 and Class 2 CRISPR-Cas systems lies in the composition of the effector complex. Class 1 consists of a multi-subunit effector complex that binds to and cleaves the target DNA or RNA, containing multiple Cas proteins. In contrast, Class 2 consists of a single, large multi-domain effector protein, such as Cas9 or Cas12, which is responsible for cleaving the target DNA or RNA (Liu et al., 2020). (i) Class 1 CRISPR-Cas system: This system is distinguished and characterized by the presence of a multi-subunit crRNA-effector complex (Ma et al., 2015). The Class 1 system includes Type I, Type III, and Type IV systems, which are primarily found in bacteria and archaea (Mohanraju et al., 1979). Most subunits of the Class 1 effector complexes, notably Cas5, Cas6, and Cas7, contain different versions of the RNA-binding RRM (RNA recognition motif) domain. (ii) Class 2 CRISPR-Cas system: This system is defined by the presence of characteristic effector modules that consist of a single multidomain protein, such as Cas9 or Cpf1. Class 2 Cas enzymes are further classified into three families: Type II, Type V, and Type VI CRISPR systems, represented by Cas9, Cas12, and Cas13, respectively (Adler et al., 2024). In addition to DNA, Cas9 and Cpf1 have been engineered to target non-coding RNAs, make base alterations, and modify epigenetic markers. Furthermore, Cas9 and Cpf1 have been designed to recognise different PAM sequences, thereby expanding their targeting capabilities (Shmakov et al., 2017).

**TABLE 2:** Classification of Cas protein of the CRISPR systems.

Class	Type	Effector module	Expression (pre-crRNA Processing)	Interference (binding & recognition)	Interference (target cleavage)	Adaptation (spacer acquisition)	Signalling/Accessory roles	Mechanism & target
Class 1	Type I	Multi-subunit (Cascade)	Cas6	Cas7, Cas5, Cas8 (LS), Cas11 (SS)	Cas3 (HD & Helicase domains)	Cas1, Cas2, Cas4	Cas4 (PAM processing)	DNA shredding: Recruitment of Cas3 nuclease
	Type III	Multi-subunit (Csm/Cmr)	Cas6	Cas7, Cas5, Cas11 (SS), Cas10 (LS)	Cas10 ((HD DNase + cOA synthesis))	Cas1, Cas2	CARF/Csm6: cOA-activated dormancy/RNA decay	Dual RNA/DNA: Transcription-coupled cleavage + signalling
	Type IV	Multi-subunit (Csf)	Unknown	Cas7, Cas5, Csf1 (LS)	Unknown	Absent	DinG: Putative helicase role	Plasmid defence: Likely DNA targeting; lacks adaptation
	Type VII	Multi-subunit	RNase III	Cas5, Cas7	Cas14 (β-CASP nuclease)	Cas1, Cas2, Cas4	Cas4	ssDNA targeting: Specifically targets single-stranded DNA.
Class 2	Type II	Single multidomain	RNase III (+tracrRNA)	Cas9	Cas9 (RuvC & HNH domains)	Cas1, Cas2, Cas4	Csn2: Likely involved in adaptation regulation	Blunt DNA cut: Direct cleavage; highly site-specific
	Type V	Single multidomain	Cas12 (Cpf1)	Cas12 (Cpf1)	Cas12 (RuvC domain)	Cas1, Cas2, Cas4	—	Staggered DNA cut: Creates 5′ overhangs (sticky ends)
	Type VI	Single multidomain	Cas13 (C2c2)	Cas13 (C2c2)	Cas13 (HEPN RNase domains)	Cas1, Cas2	—	RNA interference: Target-specific + collateral RNA decay

The table outlines the major features that distinguish Class 1 and Class 2 CRISPR–Cas systems across representative types (I, III, IV, VII, II, V, and VI). For each system, the effector architecture, expression (pre-crRNA processing), interference stages (crRNA–target binding/recognition and target cleavage), adaptation (spacer acquisition), and signalling or accessory components are indicated, together with the primary molecular mechanism and target nucleic acid. Class 1 systems employ multi-subunit effector complexes, whereas Class 2 systems rely on single multidomain effector proteins.

#### 4.3.1 Parts of the CRISPR-Cas9 system

The Cas9 protein consists of two lobes: a nuclease (NUC) lobe and a recognition (REC) lobe, as revealed by crystallographic studies. The REC lobe is made up of three regions: the REC1 domain, the REC2 domain, and an extended α-helix entitled as bridge helix. The NUC lobe comprises the RuvC, HNH, and PAM-interacting (PI) domains (Figure 6). At the interface between the REC and NUC lobes, having a positive charge groove accommodates the negatively charged sgRNA: target DNA heteroduplex. The RuvC domain has a positively charged surface that interacts with the 3′ tail of the sgRNA within the NUC lobe. This domain is composed of three separate RuvC motifs (RuvC I–III) and interfaces with the PI domain.

The HNH domain is located between RuvC II and III (Figure 6). In the REC lobe, the REC1 domain features an extended α-helical structure with two β sheets and 25 α helices, while the REC2 domain adopts a bundle structure composed of six helices. Studies have shown that the REC lobe is a functional region unique to Cas9, as it lacks structural similarities to the additional known proteins. Further research revealed that the regions connecting the HNH and RuvC domains can undergo conformational changes, allowing the HNH domain to approach and cleave the target DNA.

The PAM-interacting (PI) domain in the NUC lobe is an elongated structure made up of three α helices, five β-stranded antiparallel β sheets, two-stranded antiparallel β sheets, and seven α helices. The non-complementary DNA strand’s PAM sequence is recognized by the PI domain, dependent on the current structural emplacement of the bound complementary DNA strand (Figure 6) and the RuvC domain’s active site. The synthetic tetraloop connecting the sequences from crRNA and tracrRNA forms the sgRNA. The tracrRNA sequence can be split into three stem loops and an anti-repeat (14 nt) region, whereas the repeat (12 nt) and guide (20 nt) sections of the crRNA sequence are separated. The crystal structure illustrates that the sgRNA attaches itself to the target DNA to form a T-shaped structure that includes a repeat: anti-repeat duplex, a guide: target heteroduplex, and stem-loops 1–3. The repeat: anti-repeat duplex and stem loop 1 are connected by a single nucleotide (A51), whereas stem loops 1 and 2 are connected by a 5-nt single-stranded linker.

To form the Cas9-sgRNA binary complex, Cas9 recognizes the repeat: anti-repeat duplex and the PAM-proximal guidance region of the sgRNA. The ternary complex is formed when the binary complex identifies the DNA sequence complementary to the 20-nucleotide guide region of the attached sgRNA. The R-loop forms when the PI domain acknowledges the PAM sequence on the non-complementary strand, which occurs before the generation of the ternary complex. The strand that is complementary in the guide is cleaved by the shifting HNH domain during the tertiary complex’s assembly: target heteroduplex, while the single-stranded non-complementary strand is cleaved by the RuvC domain.

PAM recognition by Cas9 is vital for the binding and cleaving of target DNA, as supported by biochemical studies. This indicates that the Cas9-sgRNA complex undergoes an inactive-to-active conformational change following PAM recognition. The theory that the current structure likely represents an inactive state, with the HNH domain positioned away from the complementary strand, supports this idea (Sternberg et al., 2014; Gasiunas et al., 2012; Nishimasu et al., 2014).

#### 4.3.2 CRISPR- mechanism of action

The CRISPR-Cas system operates by identifying and fragmenting foreign DNA or RNA in a sequence-specific way. The immune defence mechanism of CRISPR-Cas9 can be harnessed for genetic modification and engineering applications. The Cas9 restriction enzyme or molecular scissor is guided to almost any DNA sequence in the genetic material by the engineered sgRNA or crRNA–tracrRNA structure, which is based on a customised 20-nt guide RNA sequence. After that, Cas9 is directed by this complex to cause a break in both strands (DSB) in the designated DNA. Two distinct Cas9 nuclease domains are used by host-mediated DNA repair pathways to fix the DSB.

Without a repair template, the error-prone non-homologous end joining (NHEJ) pathway is activated, often causing random insertions and deletions (indels) or even substitutions at the double-strand break (DSB) site, typically compromising gene function. If a donor template with the desired sequence and homology arms is available, the error-free homology-directed repair (HDR) pathway is initiated. This route forms the foundation for precise gene editing techniques like gene knock-ins, deletions, repairs, or mutagenesis, making it easier to produce specific mutations through homologous recombination of DNA. CRISPR-Cas9, guided by RNA for DNA binding, can be separated from its cleavage activity by altering the amino acids in the HNH and RuvC nuclease domains. This creates a versatile platform suitable for many applications beyond genome editing.

When the Cas9-sgRNA complex binds to double-stranded DNA (Figure 6), it bends the DNA by approximately 50°, flipping out the three bases adjacent to the PAM. This initial DNA bending is sufficient to facilitate further unwinding and formation of a heteroduplex. If a 3-bp mismatch occurs at the centre of the target DNA sequence, the DNA does not bend, preventing the HNH domain from adopting a catalytically active conformation. However, when the mismatch is located away from the PAM, DNA bending occurs, and the Cas9 conformation becomes catalytically competent. The acronyms for CRISPR RNA include crRNA, nucleotide, protospacer adjacent motif (PAM), single-guide RNA (sgRNA), and trans-activating CRISPR RNA (tracrRNA) (Jiang and Doudna, 2017; Gong et al., 2018; Kulishova et al., 2023).

#### 4.3.3 CRISPR-Cas system: molecular mechanism

The molecular mechanism of the CRISPR-Cas system occurs in three stages (Table 2), which collectively defend against foreign viral genetic material:

1. Adaptation (Spacer Acquisition): In this stage, the foreign genetic material’s specific sequence, known as the protospacer, is merged into the array of CRISPR, forming a new spacer. Cas1 and Cas2 proteins are primarily involved in the spacer acquisition process and found in all CRISPR-Cas types except for type IIIC, IIID, and IV systems. Spacer acquisition can occur in two forms: when the invader is encountered for the first time or when the invader already has a record in the CRISPR array. The processes of selecting protospacers and spacer creation, followed by the addition of a new repeat sequence, lead to the formation of a new spacer. Spacer deletion may also occur to regulate the CRISPR array size, although this is not well understood. Cas1 and Cas2 are critical for spacer acquisition in type I CRISPR-Cas systems, and in type I-B, Cas4 is also required for adaptation. Some systems, such as those in type I, utilise priming, where the interference mechanism helps absorb, new spacers following crRNA-guided binding to a protospacer from a prior infection (Richter et al., 2014; Wei et al., 2015; Heler et al., 2015).
2. Biogenesis: The CRISPR array is transcribed into a long precursor crRNA (pre-crRNA), which is processed into mature guide crRNAs that carry sequences corresponding to the invading pathogen. In type I and III systems, the Cas6 family proteins carry out the processing. In type I-C systems, where Cas6 is absent, Cas5d processes the pre-crRNA. In class 2 CRISPR-Cas systems, further trimming by an unidentified nuclease results in mature crRNAs that form hairpin structures (Vorontsova et al., 2015; Fonfara et al., 2016; Zetsche et al., 2015).
3. Interference: Mature crRNAs serve as guides to target and interfere with incoming nucleic acids. In class 2 systems, a single effector protein mediates target interference, whereas class 1 systems rely on Cascade-like complexes. Type III systems use the crRNA’s 5′ tag to distinguish self from non-self-sequences, ensuring that the target is only cleaved when this tag does not base-pair with the target. In type II CRISPR-Cas systems, the effector protein Cas9, guided by the tracrRNA: crRNA duplex, introduces a double-strand break in the target DNA. Type III and V systems use complexes like Cas10-Csm and Cas10-Cmr, which target both DNA and RNA, to mediate interference. Notably, interference in type III systems depends on the transcription of target DNA, and type V-B systems require a crRNA: tracrRNA duplex for target cleavage (Richter et al., 2014; Vorontsova et al., 2015; Swarts et al., 2012).

## 5 Therapeutic application

Mutations and dysregulated expression of various genes, including cancer-causing genes, tumour-suppressor genes, chemo-resistant genes, metabolism-related genes, and genes linked to cancer stem cells, are crucial for the initiation and spread of cancer. The ultimate goal of cancer treatment is to inhibit tumour growth and progression by precisely correcting these mutations and restoring the expression of dysregulated genes.

### 5.1 Therapeutic role of CRISPR

Patients with resistant lung cancer were the first to receive CRISPR-based treatment in a human trial. Researchers used CRISPR/Cas9 to alter T-cells derived from blood samples of three patients by deleting three genes (TRAC, TRBC, and PD-1) that could potentially block their progress or movement, the T-cells’ capability to fight cancer cells. Those receiving treatment were subsequently given the altered T-cells again. These modified T-cells can target and terminate cancer cells by focusing on specific antigens (Stadtmauer et al., 2020). Additionally targeting the HIV genome, CRISPR/Cas9 can be used to modify the host cell’s chemokine co-receptor type-5 (CCR5) gene, which prevents HIV from entering the cell (Table 3). An *in vitro* study conducted in China demonstrated that the researchers concluded that these altered cells were more successful in inhibiting transmission of HIV than unchanged ones after finding that CRISPR/Cas9 genetic modification of CCR5 did not cause toxicity (infection) in cells (Liu et al., 2017).

**TABLE 3:** Select approved and clinical-stage gene editing therapies in precision medicine (Clinical Trials, 2025; Wang and Cannon, 2016; FDA, 2025b).

Disease/Condition	Gene editing tool/Mechanism	Target gene/Mutation (if specified)	Key outcome/Benefit	Current status	Example therapy/Company (if applicable)
Sickle cell anemia	CRISPR-Cas9, gene replacement	BCL11A, HBB	Curative treatment restored hemoglobin production	Approved, Phase 3	CASGEVY (Vertex/CRISPR therapeutics), LYFGENIA (bluebird bio)
Beta-thalassemia	CRISPR-Cas9, gene replacement	BCL11A, HBB	Curative treatment, reduced transfusion dependence	Approved, Phase 3	CASGEVY (Vertex/CRISPR therapeutics), Zynteglo (bluebird bio)
Duchenne muscular dystrophy	CRISPR-Cas9, gene correction	DMD	Restored muscle protein function, slowed progression	Approved	ELEVIDYS (Sarapeta therapeutics)
Leber Congenital Amaurosis (LCA)	Gene editing, CRISPR-Cas9	Specific mutation	Vision restoration	Approved	LUXTURNA (Spark therapeutics)
Hemophilia B	Gene therapy	Factor IX	Reduced bleeding, potentially curative	Approved	HEMGENIX (CSL Behring)
Cerebral Adrenoleukodystrophy (CALD)	Gene therapy	​	Extended life expectancy	Approved	SKYSONA (bluebird bio)
Various cancers (e.g., lymphoma, Myeloma, AML)	CAR-T cell therapy (CRISPR-modified T cells), oncogene inactivation	Various cancer antigens, oncogenes (e.g., MYC, PD-1)	Reduced tumor growth, enhanced immune response	Approved, Phase 1/2/3	KYMRIAH (Novartis), YESCARTA (Kite Pharma), TECARTUS (Kite Pharma), ABECMA (Celgene), BREYANZI (Juno), CARVYKTI (Janssen), AUCATZYL (Autolus), AMTAGVI (Iovance)
HIV	ZFNs, CRISPR-Cas	CCR5, viral DNA	Reduced susceptible cells, viral elimination	Clinical trials (ZFNs: most advanced)	Excision Biotherapeutics therapy (CRISPR-based)
Chronic hepatitis B virus	Gene editing	Viral markers	Elimination of persistent virus	Clinical trials (early data)	Precision BioSciences therapy
High cholesterol	Gene editing	PCSK9	Cholesterol reduction	Clinical trials (early data)	YolTech therapeutics (PCSK9 silencing)
Autoimmune diseases	CRISPR-based therapies	Various	Disease modification	Clinical trials (Phase 1/2/3)	Bioray laboratories, genentech, CRISPR therapeutics, EdiGene Inc., Caribou Biosciences, Inc.
Primary hyperoxaluria type 1	Gene editing	Gene involved in oxalate production	Reduced harmful oxalate levels	Clinical trials (promising results)	YolTech therapeutics
Inherited eye disease (mutation-associated retinal dystrophy)	Gene therapy	Specific mutation	Vision restoration	Approved	Luxturna (Spark therapeutics)

By deactivating the HNH and RuvC domains, researchers created the dCas9 nuclease, a modified form of the Cas9 endonuclease. While the dCas9 nuclease does not exhibit DNA cleavage activity, it retains DNA-binding capability. By fusing dCas9 with transcriptional activators or inhibitors, the CRISPR/dCas9 complex can either silence (CRISPRi) or activate (CRISPRa) the expression of a specific gene of interest (Dominguez et al., 2015). However, because the CRISPR/Cas9 system originates from bacteria, host immunity may trigger an immune response against its components, posing a significant challenge to its therapeutic use (Charlesworth et al., 2019). Research has also shown that healthy individuals may have pre-existing immune responses to the Cas9 protein, both cellular (anti-Cas9 T cells) and humoral (anti-Cas9 antibodies). Consequently, reducing the immunogenicity of the Cas9 protein remains one of the biggest obstacles in clinical trials (Table 3).

### 5.2 Therapeutic role of TALEN

TALEN (Transcription Activator-Like Effector Nucleases) technology is used to treat a range of disease-related cell-autonomous traits, including dyslipidaemia, insulin resistance, hypoglycaemia, lipodystrophy, motor neuron death, and hepatitis C infection. While there is some potential for TALEN off-target effects, each genetic line still contains an abundance of unique genetic alterations (Ding et al., 2013). By combining precise genetic engineering technologies, for instance, zinc-finger nucleases (ZFNs), TALENs, or RNA-guided cleavage enzymes, with the reprogramming of somatic cells into induced pluripotent stem cells (iPSCs), personalised medicine has been significantly advanced. This includes gene and cell therapy approaches, which have expanded the options for disease modelling, toxicology screening, and cell replacement therapies (Hockemeyer et al., 2011).

Recessive dystrophic epidermolysis bullosa (RDEB) is a genetic disorder caused by the absence of type VII collagen protein, which results from mutations in the COL7A1 gene on chromosome 3. RDEB leads to skin blistering and a higher risk of squamous cell carcinoma (Fine and Mellerio, 2009). Restoring the deposition of type VII collagen at the dermal-epidermal junction (DEJ) through allogeneic hematopoietic stem cell transplantation or localised fibroblast transplantation can alleviate symptoms (Wong et al., 2008). In our study, we used TALENs to target and correct a specific mutation in the COL7A1 gene in primary fibroblasts from RDEB patients. Gene-corrected clones of these cells were then developed clinically significant quantities, suggesting that TALEN-based gene correction could be a viable treatment for RDEB.

### 5.3 Therapeutic role of ZFN

From its early successes in editing genes in microorganisms to its current applications in engineering hematopoietic stem cells (HSCs) and tumour-targeted T cells, genome editing technology has made significant advances, enabling targeted genetic modifications in various research areas. This has led to new strategies for genetic modification and has expanded into the field of cancer research (Li et al., 2020). The potential applications of genome-modifying technologies in cancer treatment are promising, particularly for targeting cancer-causing mutations and genes that prevent tumour growth (Vogelstein et al., 1979).

## 6 Precision medicine

Precision medicine is an innovative approach to healthcare that is fundamentally transforming how diseases are detected, prevented, and treated. This new model moves beyond traditional “one-size-fits-all” medical practices, emphasizing the tailoring of treatments to each individual’s unique biological makeup (Dzau et al., 2016). In 2011, the National Research Council of the United States proposed the concept of precision medicine as a revolutionary approach to healthcare and disease treatment (Duan et al., 2024). According to the FDA, precision medicine, also referred to as personalised medicine, tailors medical care to individual differences in genes, environment, and lifestyle. Its goal is to provide the right treatments to the right patients at the right time. The effectiveness of precision medicine depends on the accuracy of diagnosis and treatment. Next-generation sequencing (NGS), which rapidly analyzes large portions of a person’s genome, is a crucial advancement in the therapeutic use of precision medicine (Table 3) (FDA, 2025a). The National Cancer Institute of the USA describes precision medicine as a medical approach that utilizes information about a person’s genes, proteins, environment, and lifestyle to prevent, diagnose, or treat disease (NCI, 2025). International programs, such as the U.S. Precision Medicine Initiative (PMI), which was launched in 2015, demonstrate a shared commitment to advancing this field. The PMI wants to speed up the search for treatments for complicated diseases like diabetes and cancer, and provide people with individualized health information so they can take charge of their health (Naithani et al., 2021).

With the goal of creating a complex “computational learning system” or “knowledge network” designed to continually improve health interventions, this effort fundamentally depends on the safe collection, integration, and analysis of vast amounts of patient data. While often used interchangeably, phrases like “individualized medicine” and “personalized medicine” are generally considered similar to precision medicine (Precision Me dicine, 2022a; Delpierre and Lefèvre, 2023). The advancement of precision medicine technologies requires powerful computational tools, sophisticated algorithms, and real-world data sources to optimise and revolutionise the field (Vitorino, 2024). Traditional medical data management systems were not equipped to support the implementation of precision medicine, necessitating the development of enhanced data management systems and integrated databases for disease analysis (Jacobs et al., 2025). For instance, platforms that make it easier to incorporate omics, like nuclear magnetic resonance (NMR) spectroscopy for metabolomics, mass spectrometry for proteomics, RNA sequencing for transcriptomics, and next-generation sequencing (NGS) for genomes, are critical to the development of precision medicine (Vitorino, 2024). One such platform, NeDRex (Network-based Drug Repurposing and Exploration), integrates biomedical databases like OMIM (Amberger et al., 2019), DisGeNET (Piñero et al., 2020), UniProt (Bateman, 2019), NCBI gene info (Maglott et al., 2005), IID (Kotlyar et al., 2019), MONDO (Mungall et al., 2017), Drug Bank (Wishart et al., 2018), Reactome (Jassal et al., 2020), and Drug Central (Ursu et al., 2019). This integration allows the construction of variegated networks that represent different types of biological entities (e.g., diseases, genes, drugs) and the relationships between them (Sadegh et al., 2021). Similarly, bioinformatics platforms like iProClass and iProXpress enable combining and functionally annotating datasets to produce thorough analyses and significant biological insights (Kotlyar et al., 2019; Huang et al., 2003; Wanichthanarak et al., 2015).

The advancement for the development of precision medicine, which increases the efficacy and specificity, includes mainly the CRISPR-based genome editing and RNA editing tools such as CRISPR–Cas9/Cas12/Cas13 nucleases, DNA base editors, prime editors, and RNA base editors (Tao et al., 2023). With the improvement of the Cas-9 protein system, two advanced editing tool systems have been developed, which are base editing and prime editing. Base editing technology specifically allows for single-nucleotide changes such as A to G and C to T without resulting in double-stranded DNA breaks and chromosome breaks. Base editing is of two types. One is the Cytosine base editor (CBE) and another Adenine base editor (ABE), which facilitate the precise conversion of the pyrimidine bases (C to T) and purine bases (A to G) for both DNA and RNA (Kantor et al., 2020). However, Prime-editors mainly use an engineered reverse transcriptase infused with Cas9 nickase and a prime-editing guide RNA (pegRNA). pegRNA comprises the following three components: a region for binding to the RT, a template sequence used for editing the target region, and a sequence complementary to the target site to guide the directed binding of nCas9. These components, pegRNA, are the most significant part of the PE system (Shuto et al., 2024). The advancement of the BE and PE has given the CRISPR/Cas9 system new concepts and possibilities of genome editing with much more precision and specificity. Despite its effectiveness, CRISPR/Cas9 has the risk of causing gene changes because of double-stranded DNA breaks. By removing the risks connected to DNA DSBs, base editing (BE) and prime editing (PE) have reduced these risks and made it possible to edit genes more precisely. This innovation provides a strong basis for the usage of gene editing technologies in therapeutic settings (Liu et al., 2024). With the advent of next-generation technologies like base editing (BE) and prime editing (PE), recent developments (Figure 1) in genome editing have addressed significant flaws in older tools like ZFNs, TALENs, and conventional CRISPR-Cas9. Instead of traditional methods that depend on homology-directed repair (HDR) and double-strand breaks (DSBs), which are frequently ineffective, prone to errors, and limited to dividing cells, BE and PE enable precise, programmable alterations without the utility of donor DNA templates or DSBs. BE uses deaminase-fused catalytically impaired Cas9 to instantly transform single bases (C→T or A→G), allowing for highly effective point mutation repair with little off-target activity. PE extends this capacity by introducing all 12 potential base changes, tiny insertions, or deletions using a Cas9 nickase attached to reverse transcriptase, directed by a pegRNA template. These developments make BE and PE revolutionary tools for precision medicine applications that cover haematological disorders, to cardiovascular and metabolic diseases. They also greatly improve editing precision, expand therapeutic applicability across both dividing and non-dividing cells, and decrease unintended genomic alterations (Koodamvetty and Thangavel, 2025).

This proactive approach, which has been conferred in a number of ways, presents the ability to use molecular markers to detect disease risk or even its existence before obvious clinical signs appear. For example, genetic testing for changes in the BRCA1 or BRCA2 genes can direct prophylactic surgery or increased mammography frequency as preventive strategies against ovarian and breast cancer (Precision Medicine, 2022b; Precision Medicine, 2022c).

Wide-ranging effects on public health result from this fundamental shift, which may lessen the long-term burden of chronic and hereditary diseases by encouraging early intervention and facilitating accurate risk assessment. It also envisions a time when people would have more insight into their health tendencies, allowing them to make better decisions. Another emerging technology is organoid-on-a-chip, which represents a transformative milestone in precision medicine and biomedical research. The creation of organoids has been greatly enhanced by advances in microfluidic chip technology. By integrating organoid cultivation into microfluidic devices, it is easier and more accurate to control oxygen levels, nutritional gradients, and fluid flow—all critical factors for preserving the viability and functionality of organoids (Man et al., 2024).

### 6.1 Gene editing’s contribution to precision medicine: key applications

With the ability to create therapies that are precisely matched to each person’s own genetic profile, gene editing technologies are revolutionizing the treatment of a broad range of human disorders. This is a substantial advancement in the real-world implementation of precision medicine (CRISPR, 2025).

#### 6.1.1 Targeted treatment of monogenic genetic disorders

Directly repairing the underlying mutations that cause monogenic genetic illnesses, gene editing technologies are proving to be revolutionary in the treatment of these conditions. This is the “ultimate version of precision medicine,” in which treatments are specifically tailored to address a person’s unique genetic flaw (Precision Medicine, 2022d).

Sickle Cell Anemia (SCD) and Beta-Thalassemia: Major progress has been made in treating these severe blood diseases, which are brought on by mutations that impact hemoglobin manufacturing. The BCL11A gene, which codes for fetal hemoglobin, is activated by CRISPR-based medicines such as CASGEVY (exagamglogene autotemcel), which have been approved by regulatory agencies and provide a possibly curative, one-time treatment (Cetin et al., 2025). Another licensed gene editing therapy for sickle cell disease is called LYFGENIA (lovotibeglogene autotemcel) (Table 3) (Clinical Trials, 2025).

Muscular Dystrophy (e.g., Duchenne Muscular Dystrophy): The DMD gene encodes a vital muscle protein, and mutations in this gene can be fixed using CRISPR-Cas9 (CRISPR-Cas9, 2025). The FDA has approved ELEVIDYS (delandistrogene moxeparvovec-rokl) (Table 3) as a gene therapy for Duchenne (FDA, 2025b).

Cystic Fibrosis: Numerous mutations in the CFTR gene produce this hereditary disorder, which is typified by serious respiratory issues. It may be possible to modify these many mutations one at a time using CRISPR technology (CRISPR-Cas9, 2025). Base editing has been effectively employed by researchers to fix CFTR mutations *in vitro* (CRISPR, 2025).

Huntington’s Disease: Researchers are investigating using CRISPR/Cas13 to target and snip the messenger RNA (mRNA) that codes for the disease-causing mutant proteins in this neurodegenerative condition (CRISPR, 2025).

#### 6.1.2 Advancements in precision oncology

By facilitating targeted interventions against the genetic causes of cancer, boosting immune responses, and interfering with tumor development pathways, gene editing is radically changing cancer research and treatment (Chehelgerdi et al., 2024). It functions as a direct treatment modality and makes it possible to identify medication targets and cancer drivers (Youssef et al., 2024).

CAR-T Cell Therapy (Chimeric Antigen Receptor T-cell Therapy): One of the best examples of gene editing in immunotherapy for cancer. A significant step toward precision medicine is represented by the combination of CAR-T and genomic treatments, which go beyond symptomatic treatment to causal, patient-specific molecular interventions (Song et al., 2024). In this procedure, a patient’s T cells are isolated, genetically engineered (usually with CRISPR-Cas9) to express a Chimeric Antigen Receptor (CAR) that selectively identifies antigens on cancer cells; these altered T cells are then multiplied, and the patient is reinfused with them (Precision Medicine, 2022d). This improves the T cells’ capacity to recognize and eliminate cancerous cells (Chehelgerdi et al., 2024). The FDA has approved a number of CAR-T cell therapies for a range of hematologic malignancies, including KYMRIAH, YESCARTA, TECARTUS, ABECMA, BREYANZI, CARVYKTI, AUCATZYL, AMTAGVI, and TECELRA (Table 4) (CRISPR-Cas9, 2025; Youssef et al., 2024). Additionally, improvements in site-specific genomic integration and “armored” CAR-T designs provide a refined route for long-term therapeutic stability and precise immunomodulation, essentially “rebooting” systems that have failed conventional pharmacological treatments (Simic et al., 2024).

**TABLE 4:** Approved CAR T-Cell therapies and developmental stages (Bhaskar et al., 2024; CAR-T Cell, 2025; CA R-T Updates, 2025, 2025).

Product name (generic)	Target antigen	Primary indications (approved as of 2025)	Costimulatory domain	Approval year	Manufacturer
Kymriah (tisagenlecleucel)	CD19	Pediatric/Young adult B-ALL; adult R/R DLBCL, HGBCL, tFL, FL	4-1BB	2017	Novartis
Yescarta (axicabtagene ciloleucel)	CD19	Adult R/R DLBCL, PMBCL, HGBCL, tFL, FL; second-line LBCL	CD28	2017	Kite/Gilead
Tecartus (brexucabtagene autoleucel)	CD19	Adult R/R Mantle Cell Lymphoma (MCL); adult R/R B-cell precursor ALL	CD28	2020	Kite/Gilead
Breyanzi (lisocabtagene maraleucel)	CD19	Adult R/R LBCL, DLBCL, PMBCL, FL, FL grade 3B; adult R/R MZL (December 2025)	4-1BB	2021	BMS/Juno
Abecma (idecabtagene vicleucel)	BCMA	Adult R/R multiple Myeloma (after 4+ lines of therapy)	4-1BB	2021	BMS/Bluebird
Carvykti (ciltacabtagene autoleucel)	BCMA	Adult R/R multiple Myeloma (after 4+ lines of therapy)	4-1BB	2022	Janssen/Legend
Aucatzyl (obecabtagene autoleucel)	CD19	Adult R/R B-cell precursor acute lymphoblastic leukemia (ALL)	4-1BB	2024	Autolus

*In vivo* genome editing has entered the therapeutic domain to treat metabolic and coagulation problems by permanently altering the patient’s native genetic code, while adoptive cellular treatments have revolutionized haematology.

A Phase 1 study (CTX310) was published in the New England Journal of Medicine in November 2025. The safety and effectiveness of a single-dose CRISPR/Cas9 treatment that targets the hepatic ANGPTL3 gene were evaluated in this study. Endothelial lipase and lipoprotein lipase are inhibited by the well-known lipid metabolism regulator ANGPTL3. The objective of CTX310 is to pharmacologically produce this protective phenotype, since people with naturally occurring loss-of-function mutations in ANGPTL3 have much reduced levels of LDL cholesterol and triglycerides (Radcliffe Cardiology, 2025; Laffin et al., 2025). In order to deliver Cas9 mRNA and guide RNA precisely to the liver, the experiment used a lipid nanoparticle (LNP) delivery technology. Adults with uncontrolled dyslipidemia who were using maximally tolerated lipid-lowering medication were the participants (Radcliffe Cardiology, 2025). The decrease in ANGPTL3 protein levels attained with a single infusion raises the possibility that *in vivo* genome editing might supplement or replace long-term drug regimens for lowering cardiovascular risk. Even while it was not immediately categorized as a dose-limiting adverse effect, the unexpected fatality that occurred almost 6 months after treatment emphasizes the need for thorough long-term monitoring in the field of genetic medicine (Laffin et al., 2025).

The loss of non-integrating vector genomes during cell division has historically hampered long-term durability in gene therapy; this issue is especially troublesome in juvenile patients’ developing livers. To address this, a crucial work that was published in Blood (2019; 133:2745-2752) used CRISPR/Cas9 to integrate a human Factor IX (hFIX) transgene site-specifically (Wang et al., 2019; Wang et al., 2020) A dual adeno-associated virus (AAV) vector method was used by the researchers. A codon-optimized hFIX cDNA with the hyperactive “Padua” mutation and homology arms for the murine albumin (mAlb) locus was included in the donor vector, whereas one vector produced SaCas9 under a liver-specific promoter. By focusing on the albumin region, the transgenic may take advantage of the native albumin promoter’s high transcriptional activity, guaranteeing strong protein synthesis (Wang et al., 2019; Wang et al., 2020).

In contrast to typical episomal AAV gene treatments, this work showed that CRISPR-mediated integration into a highly expressed locus may produce therapeutic amounts of clotting factor that survive hepatocyte proliferation. This implies that sustained genetic integration may be necessary for “true cures” for monogenic illnesses to guarantee long-term effectiveness (Lara-Navarro and Jaloma-Cruz, 2022; Pipe et al., 2019).

Inactivating Oncogenes: Oncogenes, which are frequently altered or overexpressed in cancer cells and promote unchecked cell growth and proliferation, can be specifically disrupted by gene editing (Precision Medicine, 2022d). In animal models of lymphoma, for example, it has shown that deactivating the MYC oncogene inhibits the formation of tumors. This approach targets the genetic causes of cancer in an effort to stop its growth (CRISPR-Cas9, 2025; Chehelgerdi et al., 2024).

Enhancing Immune Responses: Gene editing can alter immune cells to increase their anti-tumor action in addition to directly attacking cancer cells. For instance, T cells’ capacity to target and eliminate cancer cells can be improved by using CRISPR-based gene editing to eliminate or reduce the expression of the PD-1 protein on T cells (Chehelgerdi et al., 2024). Additionally, to counteract immunosuppression in the tumor’s surrounding environment and enhance CAR-T cell stability and functioning, especially in solid tumors, the combination of CAR-T cell treatment and immune checkpoint suppressors is being investigated (Youssef et al., 2024).

#### 6.1.3 Applications in infectious diseases and other complex conditions

The utility of gene editing extends beyond genetic and oncological disorders to a broader spectrum of complex diseases, demonstrating its versatility in precision medicine.

HIV (Human Immunodeficiency Virus): The use of CRISPR technology to directly break the viral DNA that HIV incorporates into the host’s immune system is being investigated (World Economic Forum, 2025). ZFNs, the most clinically established technology for this indication, were used in the early human genome editing applications for HIV to alter the CCR5 gene (Wang and Cannon, 2016).

Chronic Hepatitis B Virus (HBV) Infection: With encouraging decreases in viral indicators, gene editing treatments are being developed to eradicate persistent strains of the Hepatitis B virus that are resistant to existing drugs (News, 2025).

High Cholesterol: In order to enable more liver cell cholesterol receptors to eliminate cholesterol from the bloodstream, therapies that use gene editing to silence the PCSK9 gene are being explored. This could provide a one-time therapy option as an alternative to daily medications or frequent injections (The Future of Genomics, 2025).

Autoimmune Diseases: Numerous clinical trials for autoimmune disease, such as systemic lupus erythematosus, multiple sclerosis, and lupus nephritis, are currently underway, and gene editing is being studied for a number of these conditions (Clinical Trials, 2025).

Bacterial Infections: The possibility of CRISPR-based treatments for managing bacterial infections, such as urinary tract infections and *E. coli*, is also being investigated (Clinical Trials, 2025).

COVID-19: CRISPR technology is being investigated for therapeutic uses, like as stopping the virus from attacking lung cells and limiting the immune response that results in severe illness. As well it also been quickly modified for quick diagnostic testing for COVID-19 (CRISPR, 2025; World Economic Forum, 2025).

## 7 Challenges and considerations of precision medicine through gene editing

The problems with genetic modification are not distinct; rather, they are intricately linked. Technical challenges, including mosaicism, off-target effects, and inefficient distribution, have a direct effect on the safety and effectiveness profiles of treatments, which in turn affect public confidence and regulatory authorization procedures. The high price of these treatments is mostly caused by their specialized and complex production and delivery processes, which further raise considerable hurdles to access (Table 5).

**TABLE 5:** Key challenges and mitigation strategies in gene editing for precision medicine (Gene Therapy, 2025; Gene Therapy Development Services, 2025; Ates et al., 2020; Ethical Concerns of Genome Editing, 2025; ASGCT, 2025).

Challenge category	Specific challenge	Description/Impact	Current mitigation strategies/Future directions
Technical	Off-target effects	Unintended genomic modifications at non-target sites, potentially leading to adverse outcomes	Improved gRNA design, high-fidelity Cas9 variants, Cas9 nickases, base/Prime editing
	Mosaicism	Presence of both edited and unedited cells in an individual, leading to unpredictable effects or incomplete disease correction	Clonal cell selection (*ex vivo*), improved *in vivo* delivery methods to ensure uniform editing
	Delivery challenges	Inefficient or unsafe delivery of gene editing components to target cells/Tissues, especially for systemic applications	Development of novel viral (e.g., optimized AAVs) and non-viral vectors (e.g., lipid nanoparticles, hybrid systems), advancements in in vivo editing
	Immunogenicity	Host immune response against gene editing components (vectors, transgene products), leading to reduced efficacy or elimination of edited cells	Vector modification (capsid engineering, genome modification), immunosuppression, tolerance induction, personalized gene therapy
Economic & access	High cost of therapies	Exorbitant upfront prices (millions of dollars per dose) limit accessibility for most patients and healthcare systems	Value-based agreements, long-term cost-benefit analysis (e.g., lifetime savings), manufacturing streamlining, “off-the-shelf” products
	Equitable distribution/access	Risk of exacerbating social inequalities, creating a “genetic elite” if treatments are only for the affluent	CMS access models (e.g., CGT access model for Medicaid), policy reform, addressing diversity in research and clinical trials
	Scalability of manufacturing/Supply chain	Complex, patient-specific manufacturing processes and stringent logistics hinder widespread production and distribution	Outsourcing to CMOs, end-to-end integrated supply chain solutions, AI-driven process optimization
Ethical, Legal & Societal (ELSI)	Patient privacy/Data security	Highly sensitive genetic data risks discrimination, privacy breaches, and misuse	Robust data security measures (encryption, access controls), anonymization/Pseudonymization, informed consent, clear data governance, legislation (e.g., HIPAA)
	Germline editing ethics	Heritable changes to reproductive cells raise concerns about unknown long-term effects on future generations, consent, and “slippery slope” to enhancement	International consensus for clinical prohibition (currently), continued research on safety and efficacy in non-viable embryos, public dialogue, and ethical guidelines
	Social inequalities/Eugenics	Potential for gene editing to deepen societal divides or be used for eugenic purposes, reducing human diversity	Equitable access policies, strong ethical guidelines, focus on therapeutic vs. enhancement applications, disability rights advocacy
	Regulatory complexities	Novelty, uncertainty, stringent data requirements, and manufacturing hurdles slow approval pathways	Close industry-regulator collaboration, adaptive regulatory pathways (e.g., accelerated approval for rare diseases), international harmonization of standards

Therefore, overcoming the issues surrounding gene modification in precision medicine calls for a comprehensive and multifaceted strategy that combines scientific discoveries with creative policies, economic models, and strong ethical regulations to guarantee widespread and fair access (Precision Medicine, 2025; Liu et al., 2021).

### 7.1 Ethical, legal, and societal implications

Gene editing’s enormous potential raises a wide range of difficult moral, legal, and social issues that call for serious thought and continuous public discussion.

Patient Privacy and Information Security: A person’s lineage, health concerns, and possible treatment reactions can all be revealed through genetic data, which is intrinsically sensitive and very personal (Madhavan et al., 2018). Misuse or illegal access to this data may have serious repercussions, such as genetic discrimination (by insurers or employers) or privacy violations (Madhavan et al., 2018). Implementing strong security measures (such as encryption and access controls), anonymizing or pseudonymizing data whenever feasible, and restricting data sharing to what is essential are all necessary to protect patient privacy. Importantly, strict informed consent procedures are necessary to guarantee that people are fully aware of the advantages and disadvantages of sharing their data and taking part in genetic research (AMA-Code, 2025).

Germline Editing Ethics: Germline gene editing, which modifies genes in reproductive cells (eggs, sperm) or early embryos, is a significant ethical frontier. These alterations would be inherited by subsequent generations (IGI, 2025). Clinical germline editing is widely forbidden worldwide due to serious scientific, ethical, and safety concerns; however, somatic cell gene editing for the treatment of individual diseases is generally regarded as ethical (ASGCT, 2025). The “slippery slope” argument against using gene editing for non-therapeutic “enhancement” purposes, the possibility of mosaicism within the edited individual, the unpredictable long-term effects on future generations, and the difficulty of obtaining informed consent from future affected individuals are some of the main concerns (Ethical Concerns of Genome Editing, 2025).

Social Inequalities and Eugenics: A “genetic elite” with alleged advantages in health, longevity, or cognitive capacities could be created if access to pricey gene editing treatments is restricted to the wealthy. This could worsen already-existing societal disparities (Gibelli et al., 2025). This raises questions regarding the possibility that certain “undesirable” characteristics or situations could be eliminated through the use of gene editing for eugenic goals. Such a path could lessen human diversity and even lead to more prejudice toward people with disabilities (CRISPR and Beyond, 2025).

Regulatory Evolution and Safety Dynamics: Gene editing technologies confront a complicated and ever-changing regulatory environment because they are a relatively new and quickly developing subject (Gene Therapy Development Services, 2025). In comparison to the hundreds of ongoing trials, there are very few authorized gene treatments due to strict data criteria, the inherent novelty and unpredictability of these therapies, manufacturing challenges, and the high expense of the approval process (Infiniti Research, 2025). The market for CAR T-cells is expected to grow at a rate of several billion dollars by 2025, which has forced regulatory monitoring to change. The FDA switched from single-arm studies to randomized clinical trials (RCTs) as its preferred evidential standard for novel CAR T-cell treatments in December 2025. The government now anticipates that the majority of supplementary and initial approvals will show superiority over standard-of-care therapies through overall survival (OS) or other time-to-event endpoints; however, single-arm studies may still be suitable for ultra-rare malignancies (Press Release Service, 2025; Khan et al., 2024). Additionally, safety monitoring has advanced in sophistication. For patients treated with medicines that use integrated viral vectors, the FDA presently advises a 15-year follow-up to check for subsequent T-cell malignancies. The possibility of insertional mutagenesis necessitates ongoing attention, even though a recent thorough examination of more than 700 individuals revealed that secondary T-cell lymphomas are incredibly uncommon (FDA, 2025c; National Cancer Institute, 2025). Global development and access are also severely hampered by differences in national rules (Ethical Concerns of Genome Editing, 2025).

## 8 Future directions and emerging technologies

The field of gene editing is known for its constant evolution, with new techniques that surpass the capabilities of conventional CRISPR-Cas9 and promise improved precision, adaptability, and safety.

### 8.1 Advancements in delivery systems

A lot of research is being done to create safer and more effective ways to deliver gene editing components to target cells, but effective distribution is still a major barrier.

Improved Viral Vectors: To improve safety, effectiveness, and specificity—including decreased immunogenicity and higher specificity—next-generation lentivirus and adeno-associated virus (AAV) vectors are being developed (Gene Delivery Technologies, 2025)These developments are intended to overcome restrictions such as possible insertional mutagenesis and restricted packing capacity (Ates et al., 2020).

Non-Viral Vectors: Because of their decreased immune susceptibility, enhanced safety profiles (being biodegradable and non-toxic), and potential for improved targeting to particular cells or tissues, nanoparticles—including lipid nanoparticles (LNPs), polymeric nanoparticles, and inorganic nanoparticles—are emerging as promising substitutes (Ates et al., 2020; Advanced Delivery Systems, 2025). According to recent clinical trials, LNPs, for example, have shown promise in in vivo gene editing by effectively transporting ribonucleoproteins (RNPs) into cells and altering tissues (Gene Delivery Technologies, 2025; In Vivo Gene Editing, 2025).

Hybrid Systems: To increase gene delivery, strategies that incorporate the advantages of both viral and non-viral components are being explored. Examples include non-viral vectors designed with viral targeting sequences and viral nanoparticles that use viral proteins to improve delivery. The goal of these hybrid systems is to combine the safety and scalability of non-viral techniques with the high efficiency and specificity of viral vectors (Gene Delivery Technologies, 2025; Advanced Delivery Systems, 2025).

*In Vivo* Gene Editing: Developing techniques for gene editing inside the body (*in vivo*) is a prominent emphasis since it would simplify and lower the cost of *ex vivo* (outside the body) technologies. The safety and effectiveness of systemically administered CRISPR therapy have been shown by recent clinical data, which also reveal the ability to precisely target particular organs, such as the liver, following intravenous injection. This development raises the possibility of long-term remedies for illnesses that presently call for lifelong medicine (In Vivo Gene Editing, 2025).

### 8.2 Integration with AI and multi-omics data

The combination of sophisticated computer methods and extensive biological data will significantly influence precision medicine in the future. Proteomics reflects functional protein states, metabolomics sheds light on disruptions in downstream pathways, transcriptomic and epigenomic profiles show context-dependent gene regulation, and genomic data uncover causative variations and mutational landscapes. A systems-level knowledge of disease processes is made possible by integrating these complementary data layers, which makes it easier to identify actionable therapy targets and stratify patients more accurately. By making it possible to analyze high-dimensional multi-omics information to forecast disease progression, medication response, and ideal genome-editing targets, artificial intelligence (AI) and machine learning techniques are concurrently revolutionizing precision medicine (Srivastav et al., 2025). AI-driven models can forecast off-target consequences, rank prospective genes for CRISPR-based intervention, and direct the creation of customized editing plans based on unique molecular profiles. A paradigm shift toward genuinely customized, data-driven treatments is represented by the confluence of multi-omics integration, AI-assisted analytics, and precise genome editing technologies like CRISPR, base editing, and prime editing (Testa and Musunuru, 2023).

Artificial Intelligence (AI) and Machine Learning: AI is transforming many facets of precision medicine, including patient classification, drug design, and target identification.

While AI-based quality control systems may identify production irregularities in real-time, cutting waste and guaranteeing product quality, machine learning algorithms can predict material requirements with previously unheard-of accuracy. In order to minimize problems, increase patient access, and cut expenses, AI can also provide substitute solutions. AI integration has the potential to significantly improve the scalability of tailored treatments (Pharmaceutical Technology, 2025).

Multi-Omics Integration: “Big data” from a variety of sources, such as genetic, molecular, phenotypic, clinical, and digital signatures, is essential to precision medicine (Dzau et al., 2016). To develop a more thorough understanding of disease causes and individual responses, advances in gene editing will progressively combine with other “omics” technologies (such as proteomics, metabolomics, and epigenomics) (Naithani et al., 2021). The creation of more accurate biomarkers for screening, diagnosis, prognosis, drug development, and treatment response prediction will be made possible by this comprehensive data integration (The Future of Genomics, 2025).

## 9 Problems and future directions

Genome editing nucleases function by inducing breaks in chromosomal DNA. The primary challenge is ensuring that these breaks are precisely targeted. Once the break occurs, the cellular machinery responsible for DNA repair governs the subsequent events. DNA double-strand break (DSB) repair can proceed through two pathways. One is homology-dependent repair (HDR), which involves copying a donor sequence that matches the target, and the other is non-homology-dependent repair (NHEJ), which joins the DNA ends together, potentially causing mutations at the break site. Recent research suggests that organic molecule inhibitors of key NHEJ processes could be beneficial, although further studies are needed to develop reliable and efficient reagents. To enhance the effectiveness of homology-dependent repair, strategies such as designing the donor DNA to link it with the guide RNA and implementing specialised mechanisms for sequence insertion are being explored.

While nuclease platforms like CRISPR/Cas9 can be highly effective, not a single one provide utmost exactitude. State-of-the-art achievements in Cas9 protein modifications and guide RNA design have improved the system’s ability to distinguish between off-target sequences. To confirm the impact of a sequence modification, researchers commonly employ methods such as introducing changes in model organisms. Creating spontaneous mutations in the same gene, crossing into a clean genetic background, and being accompanied by a wild-type gene. In species with rapidly expanding populations, such as cultivated plants, ancestral genomes and phenotypes can be extensively sequenced and analysed. In certain medications, inaccurate mutations may be acceptable as long as they do not lead to new diseases.

Extensive research demonstrates that genome editing platforms have made significant contributions to the development of promising therapies for various human disorders. Among these, the CRISPR/Cas9 system has emerged as a particularly successful technology for gene editing. Genome editing technology is continually advancing, from bacteria to animals and plants. Furthermore, CRISPR/Cas9 offers a novel method for editing noncoding regions of the cancer genome, facilitating the functional investigation of previously unexplored genetic features. In recent decades (Figure 1), viral gene therapy has been optimised, and gene editing has introduced new possibilities for treating congenital diseases and malignancies. The remarkable progress in developing engineered genome-editing tools, such as ZFNs, TALENs, and CRISPR/Cas9, has translated genetic modification from a theoretical concept into practical applications.

In recent years (Figure 1), gene editing technology has found applications in human health, including in the development of synthetic drugs. By integrating biology with cutting-edge technologies and multidisciplinary approaches, gene editing also enhances agricultural practices, improving crop stress resistance and disease tolerance, making it an invaluable tool for agrarian breeding. The impact of gene editing on human gene therapy has also been significant.

CRISPR/Cas9 technology, for instance, has shown promise in fixing mutations associated with thalassemia and enhancing CAR-T cell therapies to improve resistance to blood cancers. While significant hurdles remain for the safe clinical use of gene editing technologies, these advancements offer substantial therapeutic potential for many human diseases. However, considerable challenges remain in refining and optimising gene editing technology for safe and effective clinical application.
